# PLA/Collagen/Hydroxyapatite Ternary Biocomposites for Biodegradable Bone Screw Applications

**DOI:** 10.3390/polym18081005

**Published:** 2026-04-21

**Authors:** Ayşegül Uzuner-Demir, Rumeysa Yıldırım, Hürol Koçoğlu, Mihriban Aydoğan-Gemici, Zehra Betül Ahi, Fatih Arıcan, Olcay Mert, Güralp Özkoç, Mehmet Kodal

**Affiliations:** 1 Polymer Science and Technology Graduate Program, Kocaeli University, Kocaeli 41001, Türkiye; ayseguluzuner95@gmail.com (A.U.-D.); olcay.mert@kocaeli.edu.tr (O.M.); 2 Halavet Food Company, Gerede 14900, Türkiye; fatiharican@halavet.com.tr; 3 Department of Chemical Engineering, Kocaeli University, Kocaeli 41001, Türkiye; rumeysa.yildirim@kocaeli.edu.tr; 4 Department of Mechanical Engineering, Bolu Abant Izzet Baysal University, Bolu 14030, Türkiye; kocogluhurol@ibu.edu.tr; 5 Department of Molecular Biology and Genetic, Gebze Technical University, Kocaeli 41400, Türkiye; maydogan@gtu.edu.tr; 6 Kazlicesme R&D Center and Test Laboratories, Istanbul 34956, Türkiye; zehraahi@kazlicesme.com.tr; 7 Department of Chemistry, Kocaeli University, Kocaeli 41001, Türkiye; 8 Xplore Instruments B.V., 6135 KT Sittard, The Netherlands; 9 Department of Chemistry, Istinye University, Istanbul 34396, Türkiye

**Keywords:** poly(lactic acid), collagen, hydroxyapatite, biodegradable bone screw, orthopedic fixation, micro-compounding, injection molding

## Abstract

Poly(lactic acid) (PLA)-based biocomposites incorporating collagen (COLL) and hydroxyapatite (HA) were produced via melt micro-compounding and subsequent injection molding. 1,4-phenylene diisocyanate (PDI) was employed as a compatibilizer, while poly(ethylene glycol) (PEG) was used as a plasticizer. The morphological, thermal, rheological, and mechanical properties, as well as surface wettability, degradation behavior, and cytotoxicity, were comprehensively evaluated. SEM and DSC analyses revealed the phase distribution and thermal transitions, while rheological measurements showed that PEG reduced melt viscosity by increasing chain mobility. Mechanical performance was evaluated using tensile, impact, and DMA tests on standard specimens, indicating that HA primarily enhanced stiffness (elastic modulus), whereas PEG improved toughness, resulting in higher impact strength. Biodegradable bone screw prototypes were produced with the same formulations and subjected to torsion, enzymatic degradation, and MTT cytotoxicity tests. Degradation results indicated that biocomposites containing PEG, collagen, and HA exhibited accelerated mass loss. Overall, the 70/20/10 PLA/COLL/HA/PEG/PDI formulation was more suitable for soft (trabecular) bone tissue, while the 70/10/20 PLA/COLL/HA/PDI formulation showed advantages for hard (cortical) bone tissue applications.

## 1. Introduction

Biodegradable polymers have attracted considerable attention in biomedical applications, particularly in tissue engineering and temporary fixation devices. Among these materials, poly(lactic acid) (PLA) is one of the most widely used aliphatic polyesters due to its biodegradability, biocompatibility, environmental friendliness, and ease of processing [1,2,3]. PLA has been widely employed in biomedical applications, including bone fixation; bone screws for the foot, knee, and hand; staples and pins for tissue fixation; soft tissue implants; fixation devices; tissue scaffolds; wound-dressing materials; and related resorbable medical devices [4]. However, its inherently brittle nature, low impact strength, and limited biological interactions restrict its direct use in load-bearing applications [2,3]. Therefore, various strategies have been developed to enhance the mechanical performance and biological functionality of PLA-based materials.

The extracellular matrix (ECM) of connective tissues is a complex mixture composed of various protein families that provide structural integrity and regulate diverse physiological functions. The ECM is defined by the supramolecular organization of fibrillar and microfibrillar networks, as well as soluble proteins, glycoproteins, and numerous other molecules, together with the resulting biophysical properties [5]. Collagen (COLL), the most abundant component of the ECM, is a fundamental structural protein widely present in all animals. In the human body, collagen accounts for approximately one-third of the total protein content and constitutes nearly 75% of the dry weight of skin [6]. COLL plays a pivotal role in regulating cellular responses, including adhesion, attachment, proliferation, and differentiation, thereby facilitating tissue regeneration [7,8,9]. Owing to its high biocompatibility and biological activity, COLL is widely used to improve the biological performance of polymeric systems. However, the low mechanical strength of COLL and its weak interfacial interactions with the PLA matrix can limit the overall mechanical performance of such composites. Recent studies have shown that the combined use of collagen and hydroxyapatite can create a biomimetic environment that closely resembles natural bone tissue, thereby enhancing biological performance [9,10,11].

Hydroxyapatite (Ca_10_(PO_4_)_6_(OH)_2_, HA), one of the main constituents of natural bone, can increase the local Ca^2+^ concentration, which in turn activates osteoblast proliferation and promotes the growth and differentiation of mesenchymal stem cells (MSCs) [12]. It is widely used in bone repair applications due to its non-immunogenic properties, non-inflammatory behavior, biocompatibility, bioactivity, excellent bone conductivity, and osteoconductive and/or osteoinductive characteristics [13,14]. Many modern bone implants are coated with HA, as it has been suggested to promote bone formation (osteogenesis) [15]. These properties have been widely associated with the ability of HA to enhance osteointegration and support bone tissue regeneration in recent studies [16]. The incorporation of HA into the PLA matrix has been shown to improve not only biocompatibility but also physical properties such as mechanical strength and stiffness, making it advantageous for bone tissue engineering applications [17,18]. In this context, PLA-based biocomposites reinforced with hydroxyapatite have recently attracted considerable attention due to their combined biodegradability, bioactivity, and mechanical performance suitable for bone tissue engineering applications [16,19]. However, the addition of rigid mineral particles can increase the brittleness of the material and adversely affect its processability. Therefore, the component ratios in multiphase biocomposites need to be optimized.

To overcome these limitations, compatibilization and plasticization strategies are widely employed in multicomponent polymer biocomposites. Reactive compatibilizers, such as diisocyanates, can enhance interfacial adhesion by promoting chemical interactions between the polymer matrix and bioactive filler phases [20]. This approach is particularly important in PLA/COLL/HA biocomposites comprising both organic and inorganic phases, as interfacial compatibility strongly influences mechanical performance and degradation behavior. In addition, plasticizers such as poly(ethylene glycol) (PEG) increase polymer chain mobility by weakening intermolecular interactions, thereby significantly reducing brittleness while enhancing the toughness and processability of the material [21,22,23]. The combined use of compatibilizers and plasticizers enables the tuning of both mechanical properties and degradation behavior in PLA-based biocomposites.

Biodegradable polymer-based bone screws have attracted increasing interest as a strong alternative to metallic fixation devices, as they eliminate the need for a secondary surgical removal procedure following the healing process [24,25]. PLA and its derivatives are well suited for these applications due to their biodegradability and widespread clinical use. From a manufacturing perspective, micro-compounding and injection molding enable the scalable production of polymer-based medical devices with complex geometries.

In this study, PLA-based biocomposites containing collagen (COLL) and hydroxyapatite (HA) were compatibilized with 1,4-phenylene diisocyanate (PDI) and/or plasticized with PEG, and processed under fixed processing conditions using micro-compounding and injection molding. The effects of variations in component ratios on the morphological, thermal, rheological, mechanical, and surface wettability properties of the biocomposites were investigated in detail. In addition, biodegradable bone screw prototypes were fabricated using selected formulations and evaluated by torsion testing, enzymatic biodegradation, and MTT cytotoxicity assays. In the literature, single or binary combinations of COLL, HA, PEG, and reactive compatibilizers in PLA-based biocomposites have been extensively studied; however, studies incorporating PDI-based compatibilization into PLA/COLL/HA biocomposites remain limited. Moreover, to the best of our knowledge, bone screw prototypes based on PLA/COLL/HA/PDI and PLA/COLL/HA/PEG/PDI systems, where PEG plasticization is considered together with PDI compatibilization, have not been reported. This work provides the first comprehensive report on the performance of these biocomposites with novel formulations in biodegradable bone screw prototypes.

## 2. Materials and Methods

### 2.1. Materials

Poly(lactic acid) (PLA, Luminy^®^ L130) was supplied by Total Corbion (Gorinchem, The Netherlands). According to the manufacturer’s technical datasheet, PLA exhibits a melt flow index (MFI) of 10 g/10 min (190 °C/2.16 kg, ISO 1133), a density of 1.24 g/cm^3^ (ISO 1183), a tensile modulus of 3500 MPa, and tensile strength of 50 MPa (ISO 527). The material has a melting temperature of approximately 175 °C and a glass transition temperature around 60 °C. Collagen hydrolysate (COLL) with a molecular weight of ≤2000 Da and a bulk density of 0.35 g/cm^3^ was supplied by Halavet Gıda Company (Istanbul, Türkiye). Hydroxyapatite (HA) was obtained from Kimyevi Maddeler (Ankara, Türkiye). Poly(ethylene glycol) (PEG, Mw = 8000 g/mol) and 1,4-phenylenediisocyanate (PDI) were purchased from Sigma-Aldrich (Gillingham, UK). All materials were used as received without any further purification.

### 2.2. Processing

**Figure 1:**
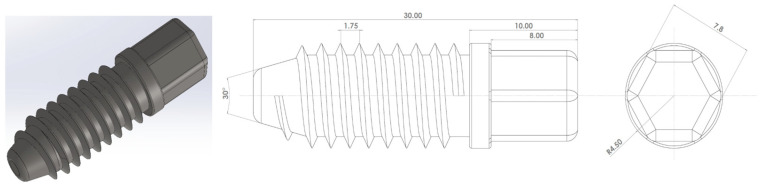
Design of a bone screw.

Before blending, PLA and HA were dried at 60 °C for 24 h, and COLL was dried at 37 °C in a vacuum oven. The compositions of all formulations were defined in terms of weight percentage (wt.%) and are summarized in Table 1. PLA/COLL/HA compositions with and without PEG were prepared using a twin-screw laboratory micro-compounder (MC15, Xplore Instruments BV, Sittard, The Netherlands). PLA, COLL, HA, PDI, and PEG were fed into the micro-compounder simultaneously. The screw speed was set to 100 rpm, the barrel temperature to 190 °C, and the residence time to 2 min. To minimize thermo-oxidative degradation, the barrel was continuously purged with nitrogen. The PDI content was kept constant at 1 wt.%, and the PEG content at 10 wt.% throughout the study. At the end of micro-compounding, the molten biocomposites were transferred from the micro-compounding nozzle to the transfer cylinder of the injection molding unit. The blends were then injection-molded using a laboratory injection molding machine (IM12, Xplore Instruments BV, The Netherlands) to produce ISO 527-5A tensile specimens, ISO 180 Izod impact specimens, and bone screw prototypes. The injection and holding pressures were set to 8 bar, and the holding time was 5 s. The melt and mold temperatures were 190 °C and 25 °C, respectively. The bone screw design, mold, and representative specimens are shown in Figure 1.

### 2.3. Characterization

Rheological behavior was assessed using an Anton Paar MCR 102 rheometer equipped with a parallel-plate geometry. Frequency sweep tests were conducted at 190 °C under a nitrogen atmosphere. The strain amplitude was fixed at 1%, and the angular frequency was varied from 0.1 to 600 rad/s.

Interfacial morphology of the biocomposites was examined using a QUANTA 400F field-emission scanning electron microscope (SEM) (Hillsboro, OR, USA). SEM images were acquired from cross-sections of impact-fractured specimens. Prior to imaging, the samples were sputter-coated with a thin gold layer to minimize charging.

Mechanical properties were evaluated by tensile and Izod impact tests. For each group, six specimens were tested, and the results are reported as mean ± standard deviation. Tensile tests were performed according to ISO 527-5A using an Instron Model 3345 universal testing machine (Instron, Norwood, MA, USA) at a crosshead speed of 10 mm/min. The Izod impact strength of unnotched specimens was measured according to ISO 180 using a CEAST Resil Impactor (Instron, Norwood, MA, USA).

Viscoelastic behavior was investigated by dynamic mechanical analysis (DMA) using a Metravib DMA 50 instrument (Limonest, France). Measurements were performed at 1 Hz with a heating rate of 1 °C/min over the temperature range of 38–120 °C.

Torsion tests were performed according to ASTM F2502-24 to evaluate the torsional behavior of the fabricated bone screw prototypes under rotational loading.

Thermal properties were analyzed using a Mettler Toledo DSC-1 STARe system (Greifensee, Switzerland) under nitrogen at a heating rate of 10 °C/min over the temperature range of 25–220 °C.

Enzymatic biodegradation of the bone screw prototypes was investigated using lipase, esterase, and alcalase to evaluate degradation in the presence of enzymes relevant to physiological environments. Samples (0.5 g) were placed in separate Erlenmeyer flasks containing 25 mL of TRIS buffer. For lipase and esterase, TRIS buffer was adjusted to pH 8.0 and 40 °C, with an enzyme concentration of 10 wt.% (relative to sample weight) and 10 mM CaCl_2_. For alcalase, samples were incubated in TRIS buffer (pH 9.5, 60 °C) with an enzyme concentration of 50 wt.% and 3 mM L-cysteine. Sodium azide (0.05 wt.%) was added to all media. Weight loss was evaluated on days 0, 1, 3, and 7. After retrieval, samples were dried in a vacuum oven at 105 °C for 90 min, cooled in a desiccator, and weighed. The percentage weight loss was calculated using Equation (1):

Weight loss (%) = [(W_1_ − W_2_)/W_1_] × 100 (1)

where W_1_ and W_2_ are the dry weights of the samples before and after enzymatic degradation, respectively [26].

Hydrophilicity properties were determined using an Attension Theta Lite contact angle measuring device (Gothenburg, Sweden). Deionized water was used as the indicator fluid in contact angle measurements.

Cell viability of the bone screw prototypes was assessed by an MTT colorimetric assay using osteoblast cells. Cells were cultured in Dulbecco’s Modified Eagle Medium (DMEM; Sigma-Aldrich, Gillingham, UK) supplemented with 10% fetal bovine serum (FBS) and 1% penicillin–streptomycin at 37 °C in a humidified atmosphere containing 5% CO_2_. Samples were placed in 96-well plates and sterilized by UV exposure for 1 h on each side. A suspension of 4 × 10^4^ cells in DMEM was seeded dropwise onto the sample surfaces and allowed to attach for 30 min at 37 °C (5% CO_2_). All samples were prepared in triplicate. Two control groups were included: a cell control (cells cultured in complete medium without any sample) and a blank control (complete medium without cells). MTT assays were performed on days 1, 4, and 7.

## 3. Results and Discussion

### 3.1. Rheological Properties

**Figure 2:**
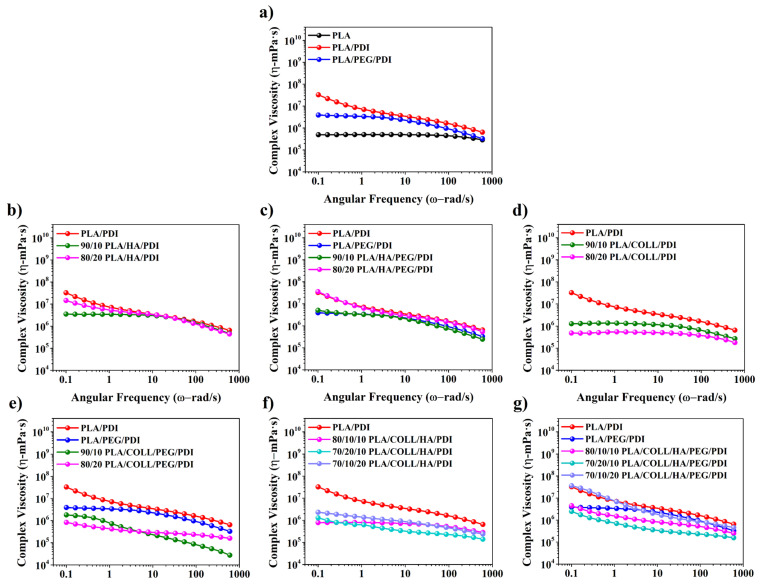
Complex viscosity versus angular frequency of PLA, PLA blends, and PLA biocomposites.

The complex viscosity (η), storage modulus (G′), and loss modulus (G″) values obtained from the rheological analyses provided important insights into the chain structure and phase interactions of the prepared PLA-based biocomposites. As seen in the complex viscosity–angular frequency curves in Figure 2, all compositions exhibited a decrease in viscosity with increasing frequency, indicating a typical pseudoplastic (shear-thinning) behavior [27]. Neat PLA exhibited lower complex viscosity values over the entire frequency range compared with the PLA/PDI and PLA/PEG/PDI blends. Upon PDI addition, a pronounced increase in complex viscosity was observed, which can be attributed to the formation of chemical bonds between the isocyanate end groups of PDI and the functional groups of PLA chains, leading to increased molecular weight and chain branching. In addition, incorporating PDI enhanced the shear-thinning behavior of PLA and shifted the Newtonian plateau toward lower angular frequencies [28,29,30]. In contrast, the addition of PEG led to a decrease in complex viscosity values by increasing chain mobility through its plasticizing effect [31]. For HA-containing composites, the complex viscosity values were generally lower than those of PLA/PDI [32]; however, a partial increasing trend in viscosity was observed with increasing filler content, which can be attributed to restricted chain mobility [33,34,35]. In collagen-containing systems, the limited compatibility between the hydrophobic PLA matrix and hydrophilic collagen led to phase separation, weak interfacial interactions, and agglomeration, resulting in reduced complex viscosity values.

**Figure 3:**
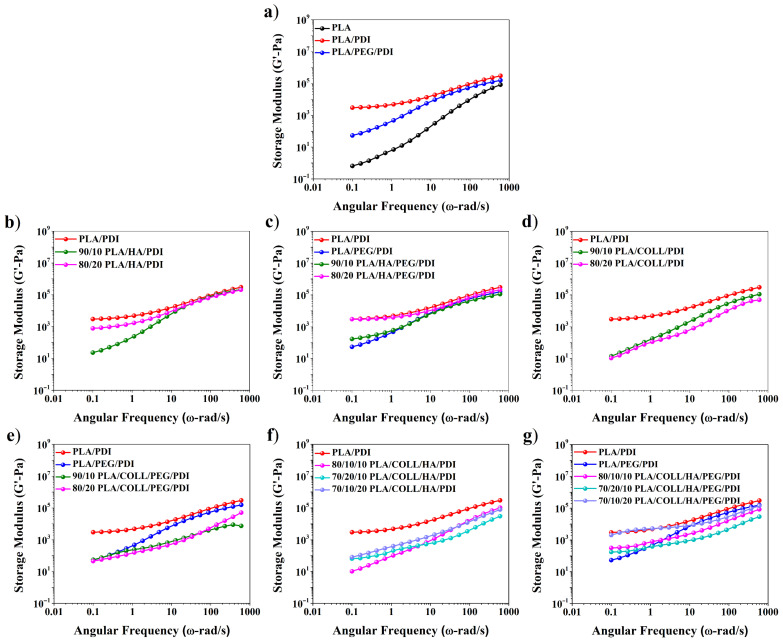
Storage modulus versus angular frequency of PLA, PLA blends, and PLA biocomposites.

**Figure 4:**
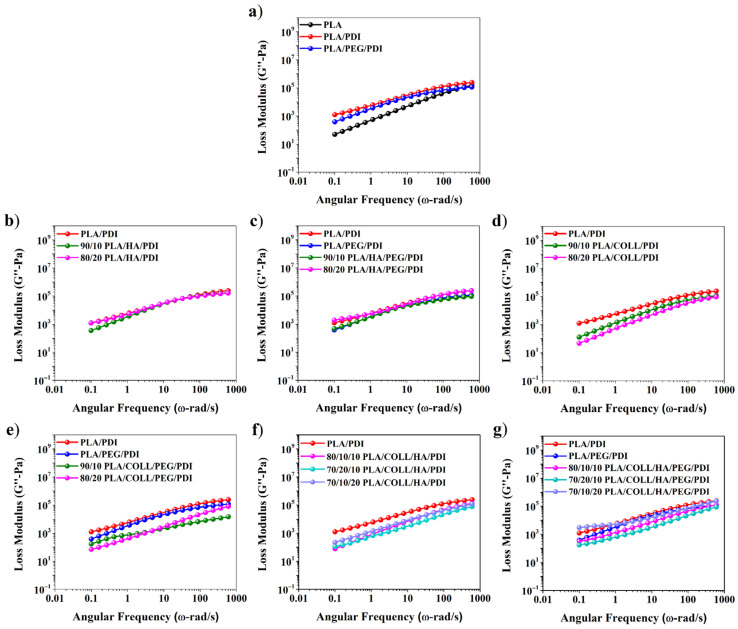
Loss modulus versus angular frequency of PLA, PLA blends, and PLA biocomposites.

As shown in Figure 3 and Figure 4, neat PLA exhibited higher G″ than G′ over the entire frequency range, indicating a predominantly viscous response. With the addition of PDI, both G′ and G″ increased; notably, the elastic response became dominant at low angular frequencies, whereas the viscous response prevailed at higher angular frequencies. This behavior can be attributed to the chain-extending effect of PDI, which enhances interchain interactions and leads to a more robust melt structure. Accordingly, PDI promoted stronger chain interactions and improved interfacial adhesion within the system, thereby increasing both the storage and loss moduli [36]. The addition of PEG, owing to its plasticizing effect, weakened chain entanglements and caused a pronounced decrease in both G′ and G″ [37], thereby making the viscous response dominant [31,38,39]. Although the incorporation of HA into the PLA matrix generally led to a decrease in the moduli due to limited interfacial interactions, partial increases were observed at higher filler contents as a result of restricted chain-segment mobility and strengthened particle–particle interactions [34,40]. This behavior can be attributed to the limited polymer–filler interactions at lower HA contents, while at higher loadings, restricted chain mobility and increased particle–particle interactions lead to an increase in the viscoelastic response. In collagen-containing biocomposites, both G′ and G″ were found to be low due to phase separation and weak interfacial interactions, resulting in a predominantly viscous behavior. This behavior suggests that collagen-induced phase separation reduces the effectiveness of stress transfer within the matrix, resulting in a weaker viscoelastic response. Overall, while PDI and PEG mainly influence chain mobility, the presence of HA and collagen affects phase interactions and the resulting viscoelastic behavior.

### 3.2. Scanning Electron Microscopy (SEM)

**Figure 5:**
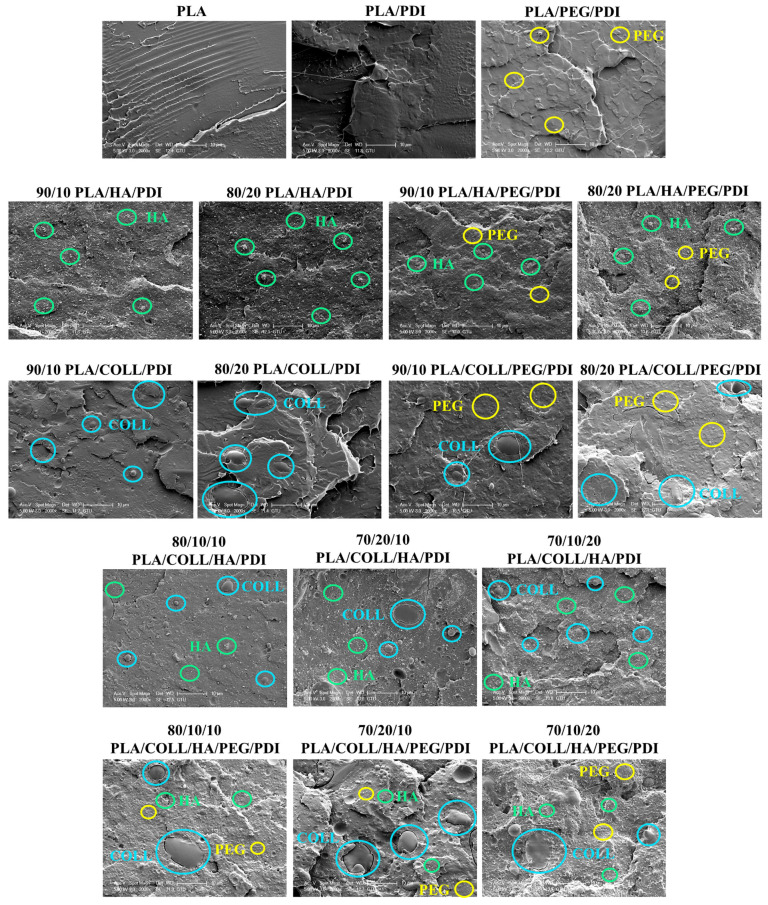
SEM images of PLA, PLA blends, and PLA biocomposites.

SEM images of the impact-fractured surfaces of neat PLA, PLA blends, and PLA-based biocomposites are presented in Figure 5. Prior to imaging, the specimens were sputter-coated with gold, and images were acquired at magnifications of 1000× and 2000×. From a microstructural perspective, the long molecular chains in PLA play an important role in elastic–plastic fracture deformation and in the crack-tip region [41]. The SEM image of neat PLA predominantly exhibited smooth surface features and brittle plastic deformation [42]. Moreover, very fine and elongated polymeric fibril-like structures were observed, and the SEM images are consistent with the low impact strength and brittle fracture behavior of PLA [43,44,45,46]. The PLA/PDI sample similarly showed a brittle fracture surface. In contrast, PEG incorporation led to a rougher fracture surface morphology, which may be associated with increased plastic deformation [47]. By weakening the intermolecular interactions between PLA chains and increasing the free volume, PEG can enhance segmental mobility, allowing the polymer chains to rearrange more easily during deformation. This effect leads to a reduction in the tensile and impact strength of PLA upon PEG addition [44,45]. The mechanical properties of biocomposites are influenced not only by the interfacial adhesion between the matrix and the filler but also by the dispersion state of the filler [48]. Hydroxyapatite (HA), a bioceramic, is inherently brittle and often exhibits a porous structure, which limits its suitability for load-bearing applications. It has been reported that HA is more homogeneously dispersed within the PLA matrix at 10 wt% [49,50], whereas increasing the content to 20 wt% results in relative agglomeration [48]. Due to the relative agglomeration of HA, premature fracture occurred in the specimens, leading to a reduction in tensile strength. These observations suggest that agglomerated HA regions act as preferential sites for crack initiation, thereby influencing the fracture behavior [51]. The rough surface morphology observed in PEG-containing PLA/HA/PDI biocomposites is associated with increased plastic deformation induced by PEG [47], which is consistent with the observed changes in mechanical properties. With the incorporation of collagen into the PLA matrix, a heterogeneous morphology was observed. Collagen appeared in the form of dispersed droplet-like domains, particularly at higher concentrations. In some regions, these domains appeared relatively well distributed within the matrix, whereas in others, larger agglomerated regions were observed [52]. In PLA/COLL/HA/PDI and PLA/COLL/HA/PEG/PDI biocomposites, both relatively homogeneous regions and localized agglomerates were observed, indicating that the dispersion of the filler phases depends on composition. In the presence of agglomeration, stress may accumulate around these regions, facilitating crack initiation during fracture. Similarly, collagen-rich domains may reduce the effective interfacial contact with the PLA matrix, creating mechanically weaker regions. These observations are consistent with the observed deterioration in mechanical performance.

### 3.3. Tensile Test Results

**Figure 6:**
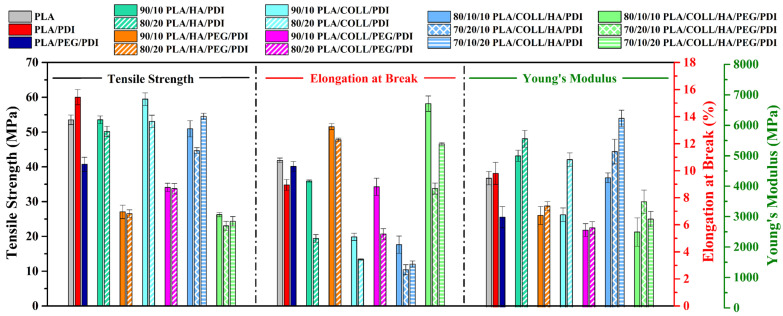
Mechanical values of PLA, PLA blends, and PLA biocomposites.

As shown in Figure 6, the tensile strength, elongation at break, and Young’s modulus of neat PLA, PLA blends, and PLA-based biocomposites are presented. Neat PLA showed brittle fracture behavior, characterized by a tensile strength of 53.5 MPa, an elongation at break of 10.7%, and a Young’s modulus of 4262 MPa [53]. The incorporation of PDI increased the molecular weight of the PLA chains, leading to higher tensile strength and Young’s modulus; however, it resulted in a decrease in elongation at break. In contrast, the addition of PEG to the PLA/PDI blend caused a pronounced reduction in tensile strength and modulus, while an increase in elongation at break was observed [53,54]. This behavior is attributed to the plasticizing effect of PEG, which weakens the intermolecular interactions between polymer chains, increases chain mobility, and enhances flexibility [21,23,47,55,56]. When the effect of HA addition on the tensile behavior was evaluated, it was found that increasing HA content led to decreases in tensile strength and elongation at break, whereas Young’s modulus increased [48,57]. This behavior can be explained by the non-uniform dispersion of HA within the PLA matrix and its tendency to agglomerate, which leads to the formation of weak interfacial interactions [58]. This indicates that HA contributes to increased stiffness as a rigid phase, while particle agglomeration at higher loadings limits load transfer and promotes earlier failure [51]. This behavior is also related to the polarity mismatch between the PLA matrix and the HA surface. Moreover, HA-rich regions limit effective stress transfer under tensile loading, contributing to earlier failure. Similarly, the incorporation of collagen led to decreases in tensile strength and elongation at break with increasing loading levels. This behavior has been associated with PLA–collagen incompatibility and phase separation. As a result, collagen-rich regions weaken load transfer under tensile loading, contributing to the observed decrease in mechanical performance. In PEG-containing systems, a significant increase in elongation at break was observed, confirming the plasticizing role of PEG in reducing brittleness and enhancing ductility. In contrast, in systems containing HA and collagen, ductility decreased at higher loadings due to restricted chain mobility and agglomeration effects. Evaluation of Young’s modulus results revealed that PEG addition caused a pronounced reduction in the stiffness of PLA [21,22], whereas the incorporation of collagen and HA increased the modulus values as a result of restricted chain mobility [48,57,58]. When the tensile strength, elongation at break, and Young’s modulus values were considered together with the mechanical requirements of bone tissues reported in the literature, the 70/10/20 PLA/COLL/HA/PDI composition was found to be more suitable for hard bone tissue applications, while the 70/20/10 PLA/COLL/HA/PEG/PDI composition showed mechanical properties closer to those required for soft bone tissue applications. Accordingly, these compositions were selected as the optimal biocomposite formulations.

Based on literature studies, the mechanical requirements for hard bone tissue include tensile strength values of 50–150 MPa [59], elongation at break values of 1–3% [60], and Young’s modulus values of 3–30 GPa [59]. In contrast, soft bone tissue is reported to require tensile strength values of 10–20 MPa [59], elongation at break values of 5–7% [60], and Young’s modulus values of 0.02–0.5 GPa [59].

### 3.4. Impact Test Results

**Figure 7:**
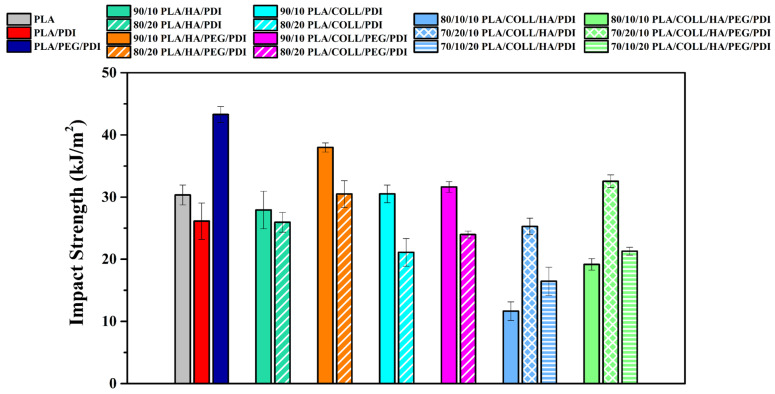
Impact strength values of PLA, PLA blends, and PLA biocomposites.

Figure 7 shows the Izod impact strength results of neat PLA, PLA blends, and PLA biocomposites. According to the impact strength data, neat PLA exhibited an impact strength of 30.35 kJ/m^2^, whereas this value decreased to 26.13 kJ/m^2^ upon PDI addition. With the incorporation of PEG into the PLA/PDI system, the impact strength increased markedly, reaching 43.29 kJ/m^2^ [53]. This increase is attributed to the plasticizing effect of PEG, which weakens the interactions between polymer chains and enhances chain segment free volume and mobility, thereby imparting a more ductile structure to the material [21,22,23]. Therefore, an increase in impact strength was observed for all PEG-containing biocomposites. When the effect of HA addition on impact strength was examined, a general decreasing trend was observed. However, for samples containing 10 wt% HA, a limited increase in impact strength was detected, likely due to the relatively homogeneous dispersion of the particles. In contrast, increasing the HA content to 20 wt% led to a decrease in impact strength as a result of the enhanced agglomeration tendency [57,61]. This behavior indicates that impact strength is highly sensitive to matrix–reinforcement interfacial interactions and nanoparticle dispersion [48]. A well-dispersed HA phase within the hybrid biocomposite can enhance the interactions between the reinforcements and the matrix; consequently, the energy absorbed by the composite during fracture may increase, which can lead to improved tensile load-bearing capacity and impact strength. However, at higher HA contents, increased particle agglomeration reduces the effectiveness of energy dissipation during fracture, leading to decreased impact strength. The addition of 10 wt% collagen to the PLA matrix improved the impact strength, whereas samples containing 20 wt% collagen exhibited reduced impact strength. At low collagen contents, a more homogeneous dispersion of the protein within the matrix increases the energy-dissipation capability, while at higher loadings, protein agglomeration leads to a deterioration in mechanical properties. As a result, the material dissipates less energy during fracture, which leads to reduced impact strength at higher collagen contents. When biocomposites containing both HA and collagen were evaluated, the impact strength results were found to be strongly dependent on the component ratios.

### 3.5. Dynamic Mechanical Testing

**Figure 8:**
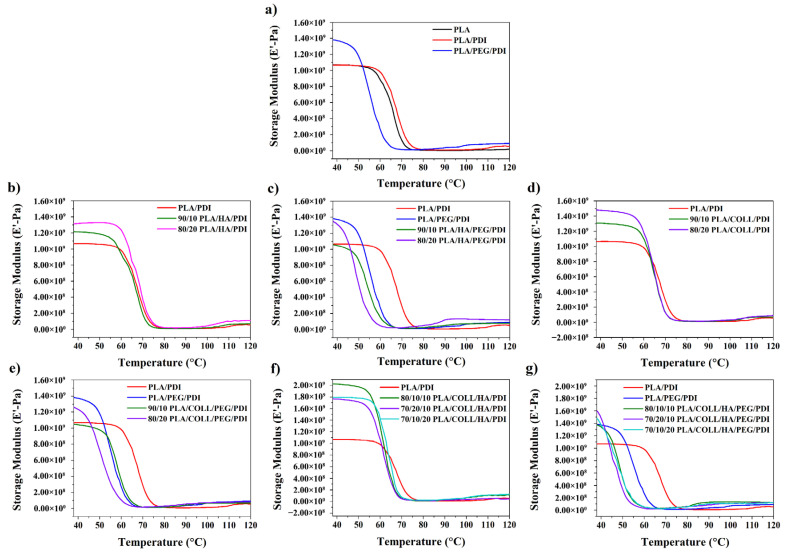
Storage modulus versus temperature curves of PLA, PLA blends, and PLA biocomposites.

**Figure 9:**
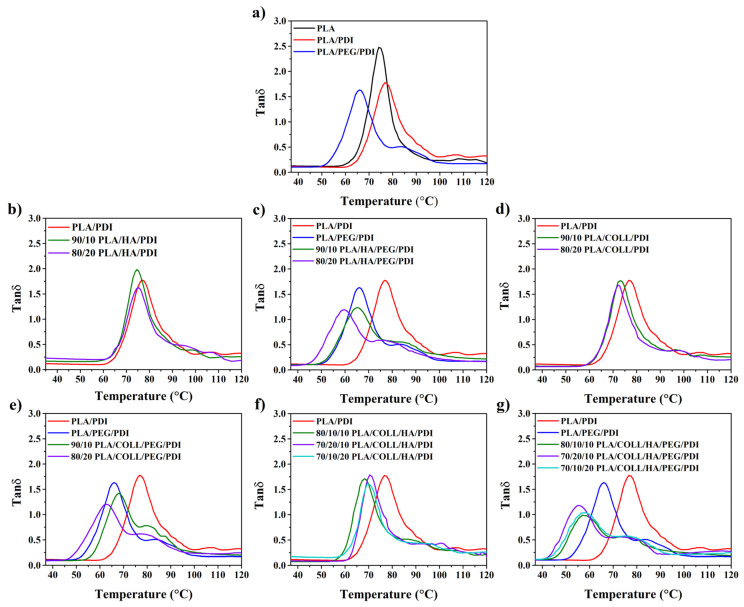
Tanδ versus temperature curves of PLA, PLA blends, and PLA biocomposites.

Figure 8 and Figure 9 show the temperature-dependent variations in the storage modulus (E′) and tan delta (tanδ) of neat PLA, PLA blends, and PLA biocomposites. The DMA results indicated that all samples exhibited similar temperature-dependent trends in storage modulus (E′). In the glassy region, the highest E′ value was observed for the PLA/PEG/PDI sample; as the temperature approached the glass transition, a pronounced decrease in modulus occurred due to increased chain mobility. Despite its plasticizing effect, PEG incorporation increased the storage modulus, suggesting an enhanced energy storage capability of the PLA/PDI system [62]. However, in PEG-containing samples, the glassy region was observed over a narrower temperature range. The incorporation of HA and collagen increased the storage modulus in the glassy region for all compositions. In particular, systems containing HA as a filler exhibited a more pronounced increase in modulus, with the highest E′ values obtained at higher HA contents [47,63,64]. Similarly, an increase in storage modulus was observed in collagen-containing samples due to restricted chain mobility. In systems where HA and collagen were used together, a synergistic effect emerged, resulting in higher storage modulus values compared with those of single-component systems. This suggests that the combined presence of HA and collagen restricts chain mobility more effectively, leading to an increased stiffness in the glassy region. For all samples, a secondary increase in E′ was observed in the rubbery plateau region at approximately 90–120 °C, which can be attributed to the cold crystallization behavior of PLA [65,66,67,68]. These results demonstrate that the fillers and additives enhance the thermomechanical stiffness of PLA-based biocomposites and that cold crystallization plays a decisive role in their mechanical behavior at elevated temperatures.

The peak positions in the tanδ curves obtained from DMA analyses reveal the glass transition temperatures (T_g_) of all samples. While neat PLA and PLA/PDI systems exhibited similar T_g_ values, a pronounced shift of T_g_ toward lower temperatures was observed upon PEG addition. This behavior is associated with the ability of PEG to increase free volume, weaken intermolecular interactions between PLA chains, and enhance chain flexibility [46]. Moreover, the presence of a single T_g_ indicates that the PEG and PLA components are miscible [62]. HA incorporation led to a decrease in T_g_ values in the PLA/PDI and PLA/PEG/PDI systems. With increasing HA content, changes in the tanδ peak height and peak breadth were observed, indicating that the relaxation behavior of the polymer chains was modified by the presence of the added components. In PEG-containing PLA/HA/PEG/PDI systems, T_g_ shifted to lower temperatures, while the tanδ peaks became lower in intensity and were distributed over a broader temperature range. According to the literature, a decrease in the tanδ peak amplitude may also be attributed to well-dispersed and well-distributed nanofillers [69]. Moreover, the shift of the tanδ peak toward higher temperatures and the reduction in peak intensity indicate increased crystallinity and the participation of fewer polymer chains in the transition [23], as well as restricted molecular mobility due to improved interactions between the filler and the matrix [67,70,71,72]. This behavior indicates that relaxation occurs over a broader temperature range in these blends due to the presence of different molecular structures [65]. Similarly, a decrease in Tg was observed in both PLA/PDI- and PLA/PEG/PDI-based systems upon collagen incorporation. Increasing collagen content led to broader tanδ peaks and reduced peak heights, indicating enhanced chain mobility and the activation of different relaxation mechanisms induced by the added components [67]. This indicates that collagen alters the relaxation behavior by introducing heterogeneous regions within the matrix, leading to a broader distribution of relaxation times. In PEG-containing PLA/COLL/PEG/PDI samples, the decrease in T_g_ was more pronounced, with lower T_g_ values obtained at higher collagen contents. A similar trend was observed in biocomposites containing both HA and collagen; Tg values were lower than those of the PLA/PDI and PLA/PEG/PDI systems in both PEG-free and PEG-containing formulations. In particular, PEG-containing PLA/COLL/HA/PEG/PDI samples exhibited tanδ peaks with lower intensities distributed over broader temperature ranges. These results indicate that the combined incorporation of the added components weakens interchain interactions, facilitates molecular relaxation, and significantly alters the thermomechanical behavior of the systems. As expected, the T_g_ values associated with the glass transition of PLA differed from those determined by DSC; however, both techniques exhibited the same overall trend [68].

### 3.6. Torsion Testing Results on Bone Screws

**Figure 10:**
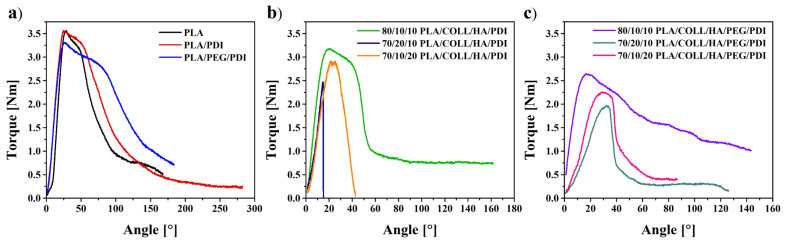
Torque versus angle of rotation curve for bone screws.

**Figure 11:**
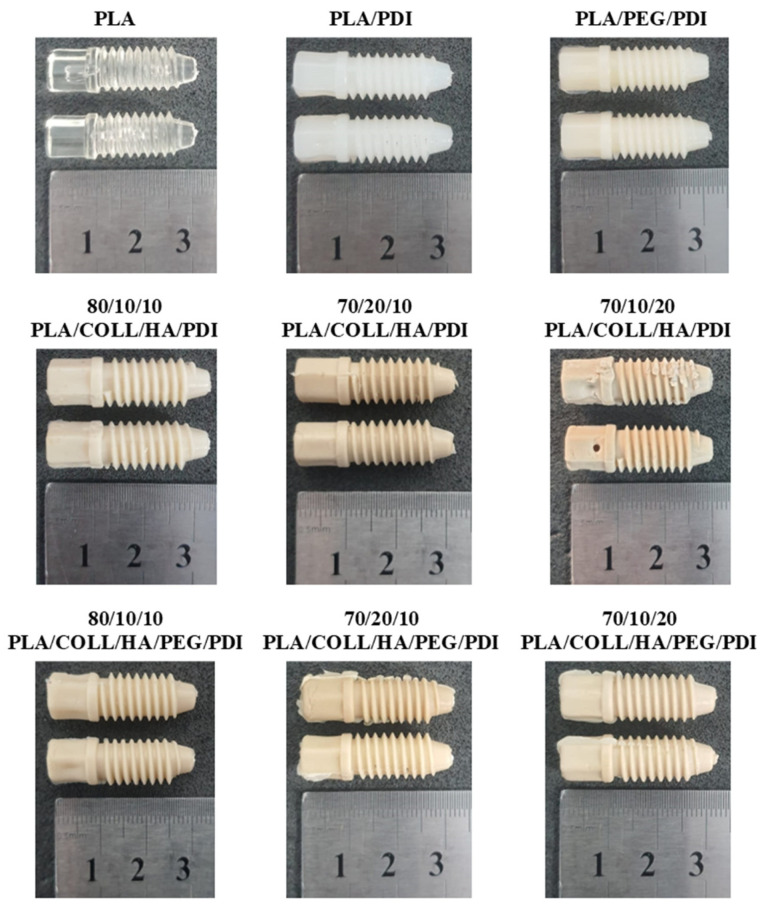
Images of bone screws before torsion testing.

The ASTM F2502 technical standard provides a standardized test method for evaluating the torsional mechanical properties of polymer screws. From a material perspective, maximum torque and fracture angle are key parameters for characterizing the resistance and deformation behavior of the material under torsional loading [73]. Figure 10 presents the torsional (torque–angle) behavior of PLA-based bone screw composites. As shown in Figure 10a, which includes neat PLA, PLA/PDI, and PLA/PEG/PDI samples, neat PLA exhibited earlier fracture and relatively brittle behavior. Moreover, Figure 11 illustrates the bone screws before torsion testing. With PDI incorporation, both the maximum torque and fracture angle increased, which is attributed to the chain-extending effect of PDI that strengthens intermolecular interactions between polymer chains. In the PEG-containing PLA/PEG/PDI sample, a slight decrease in maximum torque was observed, whereas the increase in fracture angle indicates a more ductile deformation behavior. Figure 10b illustrates the torsional behavior of PLA/COLL/HA/PDI composites containing collagen and hydroxyapatite (HA), where the mechanical response varied markedly depending on the composition. The 80/10/10 PLA/COLL/HA/PDI sample exhibited higher maximum torque values, while fracture occurred at lower angles in the 70/20/10 and 70/10/20 compositions with increased collagen and/or HA contents. This behavior can be associated with restricted polymer chain mobility and weakened interfacial interactions at higher filler loadings, leading to a more brittle structure. PLA/COLL/HA/PEG/PDI composites containing PEG (Figure 10c) generally showed lower maximum torque values but exhibited deformation over wider angular ranges, indicating a more ductile response. The plasticizing effect of PEG partially compensated for the rigid structure induced by collagen and HA, thereby enhancing the energy dissipation capacity of the material. However, compositions with high collagen content (e.g., 70/20/10) showed a pronounced reduction in torque-bearing capacity.

To provide an overall comparison, the mechanical properties are summarized in Table 2 and Table 3. In line with the torsional results, PDI enhanced tensile strength while maintaining similar torque, confirming improved interfacial interactions and chain extension. In contrast, PEG incorporation reduced tensile strength and torque but significantly improved impact resistance due to its plasticizing effect, which increases chain mobility and ductility. In PLA/COLL/HA systems, the mechanical response was strongly composition-dependent; higher HA content contributed to stiffness and strength due to its rigid, reinforcing nature, while increasing collagen content and overall COLL/HA loading led to reduced performance, likely associated with reduced interfacial compatibility and possible collagen agglomeration at higher loadings. PEG-containing biocomposites exhibited lower strength but improved toughness, indicating a transition from brittle to more ductile deformation behavior.

### 3.7. Thermal Properties

**Figure 12:**
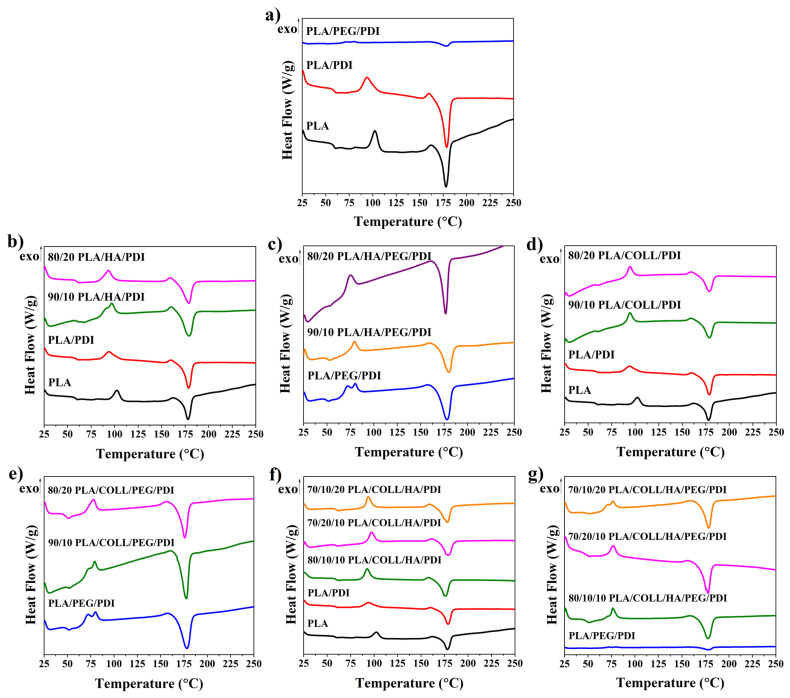
DSC curves of PLA, PLA blends, and PLA biocomposites.

Figure 12 presents the DSC thermograms of neat PLA, PLA blends, and PLA biocomposites. The glass transition temperature (T_g_) of PLA was determined as 58.7 °C, while T_g_ values of 59.1 °C and 54.7 °C were obtained for PLA/PDI and PLA/PEG/PDI, respectively (Table 4). The increase in T_g_ observed upon incorporation of the PDI chain extender into PLA indicates enhanced chain entanglement and reduced segmental mobility of PLA chains. In contrast, the addition of the plasticizer PEG to PLA/PDI resulted in a 4.4 °C decrease in T_g_. This finding suggests that PEG molecules, owing to their smaller molecular size compared to PLA chains, can readily diffuse into the matrix and significantly increase the mobility of PLA chain segments by weakening intermolecular interactions between the chains [44,46,47,74,75,76,77,78]. Moreover, this decrease in T_g_ confirms the thermodynamic miscibility of the PLA/PEG/PDI polymer blends [77]. A decrease in the melting enthalpy (ΔHₘ) was observed in the presence of PEG. This indicates that the plasticizer reduces the interfacial energy between molecules and adversely affects the crystallization nucleation of PLA [75]. Neat PLA exhibited a cold crystallization onset temperature (T_c_) of 102.4 °C, whereas T_c_ values of 93.8 °C and 85.9 °C were determined for PLA/PDI and PLA/PEG/PDI, respectively. The T_c_ of PLA/PDI blends decreased upon PEG addition, while the melting temperature (T_m_) remained nearly constant. This behavior can be attributed to the increased chain mobility induced by the plasticizer, enabling PLA to crystallize more readily at lower temperatures [44]. The incorporation of HA at different loadings into the PLA/PDI sample did not result in a noticeable change in T_g_ [79], whereas the addition of PEG to HA-containing samples facilitated molecular mobility and led to a decrease in T_g_. Similar trends were also observed in biocomposites containing collagen alone as well as those containing both collagen and HA. While the T_c_ value remained unchanged upon HA addition to PLA/PDI, PEG incorporation enhanced the crystallization ability of PLA. Likewise, the addition of HA and collagen to PLA/PDI did not alter the T_c_ value; however, the crystallization-rate-enhancing effect of PEG was observed in both PLA/HA/PDI and PLA/COLL/PDI samples. The melting temperature (T_m_) of neat PLA was determined as 177.7 °C, and no significant change in T_m_ was observed upon the incorporation of HA or collagen into the PLA matrix. Examination of the changes in crystallization enthalpy (ΔH_c_) and melting enthalpy (ΔH_m_) revealed that the melting enthalpy of HA-containing biocomposites gradually decreased with increasing HA content, indicating that HA addition hindered the nucleation and crystallization of PLA crystals. Consequently, less energy was absorbed during crystal formation [79,80]. A similar behavior was also detected in samples containing collagen and PEG. This indicates that the presence of HA and collagen interferes with the regular packing of PLA chains, thereby limiting crystal growth.

### 3.8. Biodegradation Test Results

**Figure 13:**
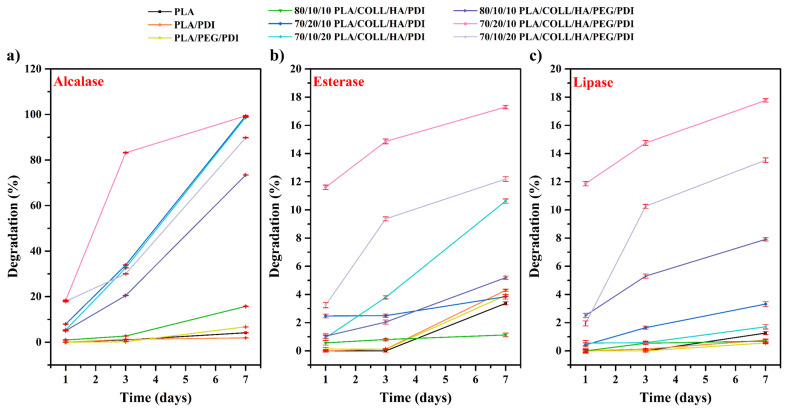
Enzymatic biodegradation test of bone screws.

The development of biodegradable aliphatic polyesters such as PLA forms the basis of advances in biomedical fields including drug delivery systems, pharmaceutical applications, and tissue engineering, where both in vivo and in vitro degradation play a critical role [81]. The ester bonds present in the structure of PLA and PLA-based biocomposites are susceptible to enzymatic degradation. During enzymatic degradation, enzymes directly catalyze the cleavage of polymeric bonds [82]. In particular, enzymes such as lipases and esterases are highly effective in cleaving ester bonds [83]. Although Alcalase is primarily a protease, auxiliary enzyme activities that may be present in its industrial formulation (e.g., esterase-like activity) can contribute to the cleavage of ester bonds in PLA. In addition, Alcalase may accelerate the degradation process by enhancing water penetration into the polymer matrix. Figure 13 presents the results of the biodegradability tests conducted using esterase, lipase, and Alcalase enzymes.

Neat PLA exhibited the lowest weight loss values, indicating a relatively stable structure under enzymatic conditions. This behavior can be attributed to the hydrophobic nature of PLA, which acts as a barrier to water uptake and enzyme penetration. In the presence of Alcalase, esterase, and lipase, the enzymes appeared to have limited penetration into the matrix; therefore, neat PLA degraded more slowly than the other biocomposites. PEG can generally accelerate degradation by increasing the water uptake capability of materials due to its hydrophilicity. However, for the PLA/PEG/PDI blend, no pronounced differences in degradation were observed under any of the three enzyme treatments. Collagen is characterized by high biological activity and good biocompatibility; nevertheless, its biodegradation rates are often higher than required [7,84].

The presence of collagen and HA can accelerate degradation under enzymatic conditions. Collagen serves as a target for proteolytic enzymes and simultaneously increases the water-holding capacity of the material, which may indirectly contribute to faster hydrolysis of PLA. In addition, HA enhances hydrophilicity and promotes the formation of a porous structure, rendering the matrix more accessible and facilitating the penetration of enzymes and water into the interior, thereby accelerating degradation [85,86,87]. When 80/10/10 PLA/COLL/HA/PDI was compared with 70/20/10 PLA/COLL/HA/PDI and 70/10/20 PLA/COLL/HA/PDI, an increase in weight loss was observed with increasing collagen or HA content. With the additional incorporation of PEG (PLA/COLL/HA/PEG/PDI), increased hydrophilicity and porosity led to high weight loss values even under lipase conditions, and the day 7 values approached 100% in some formulations under Alcalase and esterase conditions. In this context, the compositions containing 20 wt.% HA (70/10/20 PLA/COLL/HA/PEG/PDI) and 20 wt.% collagen (70/20/10 PLA/COLL/HA/PEG/PDI) exhibited the highest biodegradation rates. When comparing the enzymes, the general degradation trend for the four-component biocomposites followed the order: Alcalase > esterase > lipase. In biodegradable bone screws/scaffolds, cell–material interactions typically begin with the adsorption of soluble proteins from the culture medium onto the material surface. Over time, this protein layer undergoes rearrangement, exposing binding sites for cells and facilitating cell adhesion. PLA-based bone screws containing collagen and collagen/HA, which mimic the natural bone tissue environment, therefore exhibit strong potential to promote bone tissue regeneration as well as the adhesion and proliferation of osteogenic cells [86,88,89,90].

### 3.9. Surface Hydrophilicity

**Figure 14:**
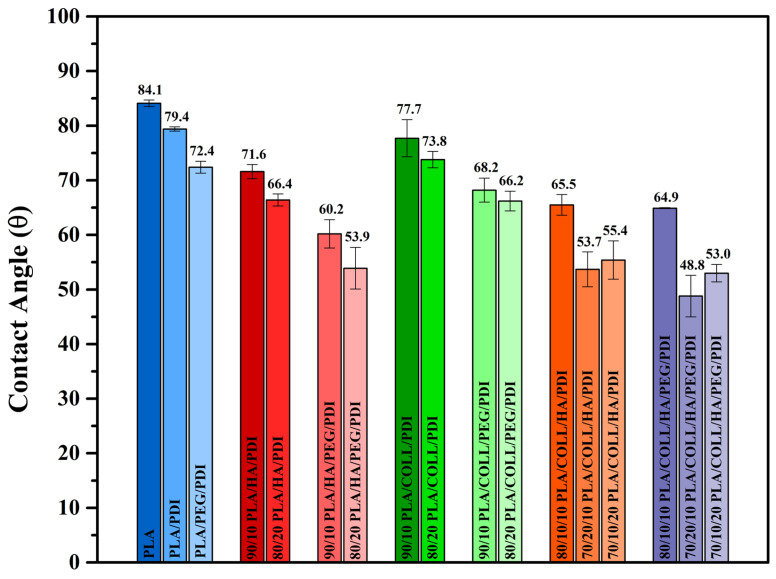
Contact angle values of PLA, PLA blends, and PLA biocomposites.

Figure 14 shows the water contact angle of a droplet on the surface of neat PLA, PLA blends, and PLA biocomposites. The hydrophobic nature of PLA limits its applicability in biomedical fields. For bone tissue materials, hydrophilicity is of great importance, as cell adhesion, migration, proliferation, and bone osseointegration are strongly influenced by the wettability of the samples [17,91,92]. A smaller contact angle indicates that the material has a more hydrophilic character [93]. A decrease in contact angle values was observed upon PEG incorporation into neat PLA (84.1 ± 0.6°) and PLA/PDI (79.4 ± 0.4°), reaching 72.4 ± 1.1°. The hydrophilic nature of PEG influenced the blend characteristics, resulting in a more hydrophilic material [93,94,95]. The contact angle values of PLA/HA/PDI biocomposites changed significantly with HA incorporation (71.6 ± 1.3° and 66.4 ± 1.1°). In particular, when the HA content reached 20 wt%, the contact angle decreased to 66.4 ± 1.1°. These results demonstrate that the addition of HA can effectively enhance the hydrophilicity of PLA-based biocomposites [17,92,96,97]. These results can be attributed to the hydrophilic nature of hydroxyapatite, which contains hydroxyl (–OH) groups [17]. The increase in hydrophilicity is expected to significantly promote HA formation by facilitating the diffusion and transport of mineral ions in the physiological environment [79]. A further decrease in contact angle values was observed upon PEG incorporation into PLA/HA/PDI biocomposites, reaching 60.2 ± 2.6° and 53.9 ± 3.8°. In PLA/COLL/PDI biocomposites, the reduction in contact angle values was found to be linear with increasing collagen content (10–20 wt%). Surface characteristics such as fiber diameter, surface roughness, and pore structure significantly influence the hydrophilicity of the material. Porosity activates bone tissue formation within the implant, thereby contributing to a faster regenerative process [98]. Therefore, the hydrophilicity of PLA/COLL biocomposites is higher than that of neat PLA (77.7 ± 3.4° and 73.8 ± 1.5°). Collagen contains hydrophilic functional groups such as –NH_2_, –COOH, and –OH; when blended with PLA, the amount of hydrophilic groups on the composite surface increases, leading to enhanced surface hydrophilicity [84]. A decrease in contact angle values was also observed upon PEG incorporation into PLA/COLL/PDI biocomposites, reaching 68.2 ± 2.2° and 66.2 ± 1.8°. With the addition of collagen and HA to PLA/PDI, the contact angle values shifted to lower levels compared to that of PLA/PDI (79.4 ± 0.4°), with values of 65.5 ± 1.9° for 80/10/10 PLA/COLL/HA/PDI, 53.7 ± 3.2° for 70/20/10 PLA/COLL/HA/PDI, and 55.4 ± 3.5° for 70/10/20 PLA/COLL/HA/PDI. This trend indicates the hydrophilicity-enhancing effect of collagen and HA. This improvement in surface hydrophilicity is particularly important for enhancing cell–material interactions and promoting osteointegration in biomedical applications. A further reduction in contact angle values was observed with PEG incorporation into PLA/COLL/HA/PDI biocomposites. The contact angle values were determined as 64.9 ± 0.1° for the 80/10/10 PLA/COLL/HA/PEG/PDI sample, 48.8 ± 3.8° for the 70/20/10 PLA/COLL/HA/PEG/PDI sample, and 53.0 ± 1.6° for the 70/10/20 PLA/COLL/HA/PEG/PDI sample.

### 3.10. In Vitro Cytotoxicity—MTT

**Figure 15:**
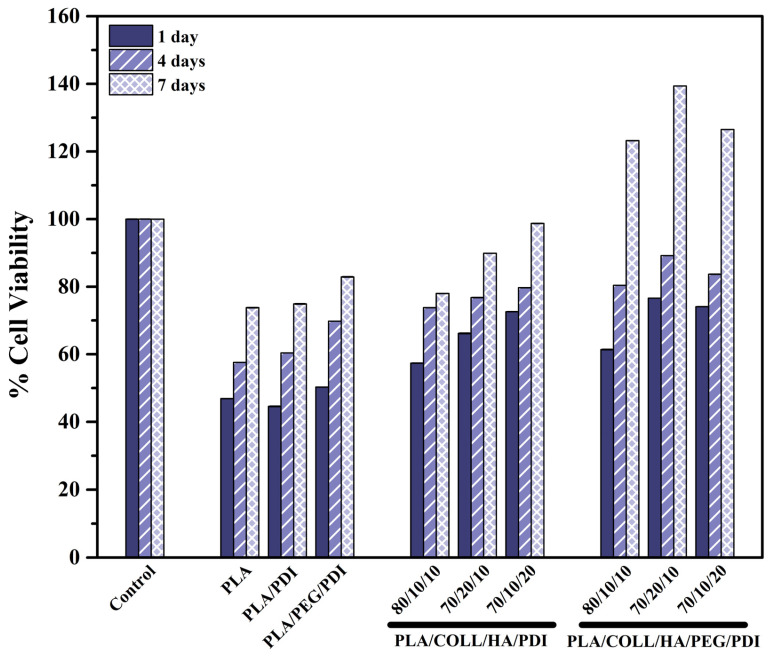
Cell viability of bone screws for osteoblast cells.

The MTT assay is a widely used colorimetric method for evaluating the effect of cytotoxicity on cell viability. The principle of the test is based on the reduction of yellow MTT to purple formazan crystals by mitochondrial succinate dehydrogenase enzymes in metabolically active cells. After dissolution of the formed formazan crystals in DMSO, the absorbance measured at 570 nm is considered to be directly proportional to the number of viable cells [99]. The MTT assay results obtained from bone fixation screw prototypes were evaluated using osteoblast cells. Figure 15 illustrates the contribution of PLA-based biocomposites to cell proliferation on days 1, 4, and 7. The relative percentage of the control cells was used to represent 100% cell viability.

For the neat PLA sample, cell viability increased from 46.9 ± 0.1% on day 1 to 73.8 ± 0.1% on day 7. In the PLA/PDI sample, the cell viability on day 7 was determined as 74.9 ± 0.1%, showing a limited improvement compared to neat PLA. This behavior suggests that PDI may support cell–material interactions through its chain-extending or interfacial-enhancing effect. In contrast, the PLA/PEG/PDI sample exhibited higher cell proliferation, reaching 82.9 ± 0.1% on day 7, compared to both neat PLA and PLA/PDI. PEG facilitates cell adhesion by providing a more hydrophilic surface environment [97]. PLA/COLL/HA/PDI biocomposites contain two important bioactive phases, namely collagen and hydroxyapatite (HA). On day 7, cell viability values of 78.0 ± 0.1%, 89.9 ± 0.1%, and 98.7 ± 0.1% were obtained for the 80/10/10, 70/20/10, and 70/10/20 PLA/COLL/HA/PDI formulations, respectively. According to these results, the formulation containing 20 wt% HA (70/10/20 PLA/COLL/HA/PDI) achieved nearly 100% cell viability on day 7, exhibiting the highest osteoblast viability among the ternary composites [100,101]. Collagen contains Arg–Gly–Asp (RGD) sequence motifs that are naturally recognized by cells, thereby supporting cell adhesion and proliferation [102]. HA is the main inorganic component of bone tissue and promotes osteogenic processes in osteoblast-like cells [101,103]. Evaluation of the day 7 results for PEG-containing PLA/COLL/HA/PDI biocomposites revealed that the highest cell viability values were obtained for the quaternary composites [97]. On day 7, cell viability values of 123.2 ± 0.1%, 139.4 ± 0.2%, and 126.5 ± 0.2% were obtained for the 80/10/10, 70/20/10, and 70/10/20 PLA/COLL/HA/PEG/PDI biocomposites, respectively. Among these formulations, the 70/20/10 PLA/COLL/HA/PEG/PDI biocomposite exhibited the highest cell viability on day 7. Increasing the collagen content to 20 wt% provided an excellent surface environment for osteoblast cells. The biocomposite with the second-highest cell viability was 70/10/20 PLA/COLL/HA/PEG/PDI, where the presence of 20 wt% HA contributed to a favorable environment supporting bone formation. Furthermore, PEG incorporation enhanced surface hydrophilicity, facilitating osteoblast adhesion to the surface. High cell viability is a positive indicator of osteoblast attachment and proliferation on implant surfaces, which may accelerate osseointegration with bone tissue. Based on these results, all samples were found to be biologically safe and exhibited no cytotoxic effects. The fact that the cell viability values of all samples exceeded the ISO-defined cytotoxicity safety threshold of 70% confirms the suitability of the developed materials in terms of cellular viability [104].

## 4. Conclusions

This study demonstrated that the mechanical, thermal, surface, and biodegradation properties of PLA-based biocomposites can be effectively tailored through the combined use of PDI, PEG, collagen, and hydroxyapatite. PDI acted as a compatibilizer and chain extender, improving melt strength and tensile performance, whereas PEG increased chain mobility, reduced brittleness, and promoted a more ductile deformation behavior. In contrast, HA contributed to stiffness and strength, while collagen and PEG enhanced hydrophilicity, biodegradation, and cellular response. A key outcome of this study is the identification of composition-dependent structure–property relationships governing the mechanical and biological performance of the developed biocomposites. Among the investigated formulations, 70/10/20 PLA/COLL/HA/PDI exhibited a more suitable property profile for hard (cortical) bone applications due to its higher strength and rigidity, whereas 70/20/10 PLA/COLL/HA/PEG/PDI showed greater promise for soft (trabecular) bone applications owing to its improved toughness, hydrophilicity, biodegradation behavior, and high cell viability. The novelty of this study lies in demonstrating that PLA/COLL/HA-based biocomposites can be tailored to meet distinct mechanical and biological requirements of hard and soft bone tissues through compositional design, providing a promising approach for the development of application-specific biodegradable orthopedic fixation materials.

## Figures and Tables

**Table 1:** Composition of PLA/COLL/HA-based biocomposites, including PEG and PDI contents, expressed in weight percentage (wt.%).

Materials	PLA (wt.%)	COLL (wt.%)	HA (wt.%)	PEG (wt.%)	PDI (wt.%)
Sample-0	100	-	-	-	-
Sample-1	100	-	-	-	1
Sample-2	90	-	10	-	1
Sample-3	80	-	20	-	1
Sample-4	90	10	-	-	1
Sample-5	80	20	-	-	1
Sample-6	90	-	10	10	1
Sample-7	80	-	20	10	1
Sample-8	90	10	-	10	1
Sample-9	80	20	-	10	1
Sample-10	100	-	-	10	1
Sample-11	80	10	10	-	1
Sample-12	70	20	10	-	1
Sample-13	70	10	20	-	1
Sample-14	80	10	10	10	1
Sample-15	70	20	10	10	1
Sample-16	70	10	20	10	1

**Table 2:** Torsional properties of bone screws.

Specimen	Max Torque [Nm]	Failure Angle [°]	Yield Torque [Nm]	Yield Angle [°]	Stiffness [Nm/°]
PLA	3.60 ± 0.05	32.07 ± 9.80	3.45 ± 0.01	24.87 ± 0.89	0.151 ± 0.007
PLA/PDI	3.62 ± 0.08	50.61 ± 13.75	3.47 ± 0.10	22.44 ± 0.00	0.170 ± 0.005
PLA/PEG/PDI	3.33 ± 0.18	46.65 ± 7.89	3.08 ± 0.10	20.55 ± 3.31	0.166 ± 0.028
80/10/10 PLA/COLL/HA/PDI	3.21 ± 0.06	40.87 ± 1.39	3.06 ± 0.07	15.78 ± 1.78	0.222 ± 0.020
70/20/10 PLA/COLL/HA/PDI	2.47 ± 1.00	14.88 ± 2.35	2.18 ± 0.81	13.08 ± 2.55	0.197 ± 0.042
70/10/20 PLA/COLL/HA/PDI	2.91 ± 0.45	32.08 ± 4.08	2.79 ± 0.43	19.47 ± 3.69	0.160 ± 0.083
80/10/10 PLA/COLL/HA/PEG/PDI	2.69 ± 0.35	34.77 ± 3.63	2.59 ± 0.31	14.25 ± 3.18	0.211 ± 0.047
70/20/10 PLA/COLL/HA/PEG/PDI	1.98 ± 0.23	35.31 ± 1.97	1.91 ± 0.17	28.38 ± 8.72	0.072 ± 0.057
70/10/20 PLA/COLL/HA/PEG/PDI	2.30 ± 0.24	37.83 ± 3.75	2.05 ± 0.29	21.99 ± 4.58	0.103 ± 0.013

**Table 3:** Mechanical properties of PLA, PLA blends, and PLA biocomposites.

Specimen	Max Torque [Nm]	Tensile Strength [MPa]	Impact Strength [kJ/m^2^]
PLA	3.60 ± 0.05	53.52 ± 1.38	30.35 ± 1.60
PLA/PDI	3.62 ± 0.08	60.02 ± 2.17	26.13 ± 2.93
PLA/PEG/PDI	3.33 ± 0.18	40.76 ± 2.04	43.29 ± 1.29
80/10/10 PLA/COLL/HA/PDI	3.21 ± 0.06	50.95 ± 2.34	11.65 ± 1.50
70/20/10 PLA/COLL/HA/PDI	2.47 ± 1.00	44.64 ± 0.87	25.27 ± 1.33
70/10/20 PLA/COLL/HA/PDI	2.91 ± 0.45	54.51 ± 0.88	16.46 ± 2.26
80/10/10 PLA/COLL/HA/PEG/PDI	2.69 ± 0.35	26.25 ± 0.61	19.17 ± 0.92
70/20/10 PLA/COLL/HA/PEG/PDI	1.98 ± 0.23	23.04 ± 1.30	32.55 ± 1.02
70/10/20 PLA/COLL/HA/PEG/PDI	2.30 ± 0.24	24.36 ± 1.35	21.30 ± 0.64

**Table 4:** DSC results of PLA, PLA blends, and PLA biocomposites.

Specimen	T_g_ (°C)	T_c_ (°C)	ΔH_c_ (J/g)	T_m_ (°C)	ΔH_m_ (J/g)
PLA	58.7 ± 0.21	102.4 ± 0.67	21.4 ± 0.77	177.7 ± 0.55	52.9 ± 0.18
PLA/PDI	59.1 ± 0.17	93.8 ± 0.91	21.0 ± 0.51	178.0 ± 0.32	55.9 ± 0.22
PLA/PEG/PDI	54.7 ± 0.23	85.9 ± 0.78	19.2 ± 0.20	180.7 ± 0.26	42.3 ± 0.26
90/10 PLA/HA/PDI	60.2 ± 0.36	96.9 ± 0.66	22.2 ± 0.30	178.7 ± 0.44	46.7 ± 0.33
80/20 PLA/HA/PDI	59.8 ± 0.28	93.4 ± 0.57	17.8 ± 0.45	178.5 ± 0.22	44.7 ± 0.19
90/10 PLA/HA/PEG/PDI	49.6 ± 0.19	80.1 ± 0.25	13.0 ± 0.18	177.8 ± 0.17	40.7 ± 0.28
80/20 PLA/HA/PEG/PDI	51.0 ± 0.22	79.3 ± 0.44	10.1 ± 0.12	179.9 ± 0.28	31.8 ± 0.34
90/10 PLA/COLL/PDI	59.7 ± 0.31	94.6 ± 0.86	22.4 ± 0.20	178.6 ± 0.48	46.8 ± 0.68
80/20 PLA/COLL/PDI	58.6 ± 0.43	95.4 ± 0.72	20.2 ± 0.13	176.7 ± 0.10	45.3 ± 0.81
90/10 PLA/COLL/PEG/PDI	51.5 ± 0.37	74.6 ± 0.43	16.1 ± 0.23	176.5 ± 0.15	40.7 ± 0.77
80/20 PLA/COLL/PEG/PDI	50.6 ± 0.22	79.1 ± 0.44	12.6 ± 0.34	177.5 ± 0.36	41.8 ± 0.63
80/10/10 PLA/COLL/HA/PDI	58.9 ± 0.28	92.6 ± 0.65	21.0 ± 0.15	175.3 ± 0.18	46.0 ± 0.44
70/20/10 PLA/COLL/HA/PDI	59.3 ± 0.19	97.2 ± 0.55	21.0 ± 0.22	178.8 ± 0.67	42.3 ± 0.52
70/10/20 PLA/COLL/HA/PDI	59.7 ± 0.24	93.8 ± 0.22	17.6 ± 0.31	177.4 ± 0.34	39.9 ± 0.37
80/10/10 PLA/COLL/HA/PEG/PDI	48.8 ± 0.33	76.5 ± 0.31	12.5 ± 0.27	177.2 ± 0.52	36.9 ± 0.35
70/20/10 PLA/COLL/HA/PEG/PDI	48.9 ± 0.11	76.8 ± 0.38	11.5 ± 0.56	177.3 ± 0.29	36.4 ± 0.26
70/10/20 PLA/COLL/HA/PEG/PDI	48.6 ± 0.17	76.2 ± 0.29	12.4 ± 0.61	178.3 ± 0.40	33.5 ± 0.32

## Data Availability

The original contributions presented in the study are included in the article, further inquiries can be directed to the corresponding author.
